# Atorvastatin combined with low-dose dexamethasone improves the neuroinflammation and survival in mice with intracerebral hemorrhage

**DOI:** 10.3389/fnins.2022.967297

**Published:** 2022-08-22

**Authors:** Yiming Song, Xuanhui Liu, Jiangyuan Yuan, Zhuang Sha, Weiwei Jiang, Mingqi Liu, Yu Qian, Chuang Gao, Zhitao Gong, Hongliang Luo, Xin Zhou, Jinhao Huang, Rongcai Jiang, Wei Quan

**Affiliations:** ^1^Department of Neurosurgery, Tianjin Medical University General Hospital, Tianjin, China; ^2^The State Key Laboratory of Neurotrauma Repair and Regeneration, Ministry of Education, Tianjin, China; ^3^Department of Neurosurgery, Xuanwu Hospital, Capital Medical University, Beijing, China; ^4^Department of Cardiology, Tianjin Medical University General Hospital, Tianjin, China

**Keywords:** atorvastatin, dexamethasone, inflammation, combination therapy, intracerebral hemorrhage

## Abstract

Intracerebral hemorrhage (ICH) is a fatal disease with high mortality and poor prognosis that triggers multiple severe brain injuries associated with an inflammatory cascade response that cannot be treated with any effective medication. Atorvastatin (ATO) suppresses inflammation, alleviates brain trauma, and eliminates subdural hematoma. Dexamethasone (DXM) also has the capacity to inhibit inflammation. Thus, we combined ATO with low-dose DXM to treat ICH mice *in vivo* to examine whether the combined treatment can inhibit secondary inflammation around the cerebral hemorrhage and decrease overall mortality. Compared to the monotherapy by either ATO or DXM, the combined treatment significantly improves the survivorship of the ICH mice, accelerates their recovery of impaired neurological function, and modulates the circulating cytokines, oxidative products, and apoptosis. Moreover, the benefit of ATO-DXM combination therapy was most pronounced on day 3 after dosing compared to ATO or DXM alone. Thus, early administration of ATO combined with low-dose-DXM promotes better survival of ICH and improves neurological function by reducing neuroinflammation and brain edema in their early phase.

## Introduction

Intracerebral hemorrhage (ICH) is the most severe kind of stroke affecting approximately 2 million people each year in the world, with an extremely high mortality of 70% at 5 years and a permanent disability rate of more than 80% (Cordonnier et al., 2018). No satisfactory results have been achieved so far to reduce the poor prognosis of ICH, either by conservative or surgical treatment (Xue and Yong, 2020).

Neuroinflammation plays a key role not only in the secondary injury after ICH but also in brain recovery. It activates microglia and other inflammatory cells and significantly upregulates multiple inflammatory mediators in the brain after ICH (Wang, 2010; Aronowski and Zhao, 2011). It is imperative to regulate the mechanism of neuroinflammation and brain edema and thus modify the pathological status of the ICH. Inhibition of the inflammatory response produces neuroprotective effects in ICH (Xu et al., 2017). While brain edema, blood-brain barrier (BBB) dysfunction, inflammatory response, oxidative stress, and cell apoptosis are involved in the pathophysiology of the secondary brain injuries of ICH (Hu et al., 2016; Bao et al., 2020; He et al., 2021), the inflammatory response plays the most prominent role in this process. Therefore, anti-inflammatory intervention in the early stages of ICH is a promising therapeutic strategy to produce neuroprotective effects and alleviate secondary brain injury.

Atorvastatin (ATO) is a hydroxy methylglutaryl coenzyme A reductase inhibitor with the potential to restrain inflammation and regulate lipid metabolism (Khurana et al., 2015). Since 2010, ATO has been tried for the treatment of ICH (Westover et al., 2011), and has been proven to help repair the BBB, restore neurological function, and inhibit inflammation and apoptosis in stroke patients (Yang D. et al., 2011; Endres et al., 2018; Saliba et al., 2018). However, clinical trials of SPARCL have shown that there may be a risk of bleeding with statins in patients with a history of stroke (Amarenco et al., 2006). Since then, it is inconclusive whether statins should be used for hemorrhagic stroke due to the lack of solid evidence of the neuroprotective effects of statins on post-stroke brain injury (Endres et al., 2018). Thus, while ATO is beneficial in hemorrhagic stroke, merely in 7–14 days after administration (Yang D. et al., 2011; Endres et al., 2018; Saliba et al., 2018), its anti-inflammatory effects are insufficient to improve the prognosis of inflammatory diseases such as ICH (Truwit et al., 2014; Boiati et al., 2015). In order to enhance the inflammation inhibitory effect of statins on ICH, we hypothesized that coadministration of ATO with another anti-inflammatory drug, such as dexamethasone (DXM), could improve neurological outcome and survival after ICH. As a synthetic glucocorticoid, DXM is a classical inflammation restraint that has been reported to be efficacious in the treatment of chronic subdural hematoma (CSDH) due to its long-lasting and powerful anti-inflammatory potentiality (Sun et al., 2005; Delgado-Lopez et al., 2009). By modulating inflammatory response, DXM allows for improved neurological function and reduced mortality in ICH patients (Zaganas et al., 2011). It is worth noting that high-dose DXM has been abandoned for the treatment of severe brain injury due to a series of complications associated with glucocorticoids, such as gastrointestinal bleeding, infection, and deterioration of diabetes (Carney et al., 2017). For example, high-dose DXM treatment (16 mg/day) could benefit adult CSDH patients with fewer repeat surgeries in the early phase of treatment, but high-dose within 6 months resulted in fewer favorable outcomes and more adverse events (Hutchinson et al., 2020). Therefore, the anti-inflammatory effects of low-dose DXM should be explored in the treatment of brain injury.

In the present study, we explored the synergistic modulatory effect of ATO and low-dose DXM on ICH mice. In order to examine their combined efficacy on neurological outcomes in the established mouse ICH model, we used survival analysis, head MRI test, behavioral tests, CBF test, brain water content test, blood-brain barrier damage examination, and inflammatory cytokine array. We found that the combined treatment was superior compared to the monotherapy. We expect that the combined treatment could improve the neurofunction and recovery of mice after ICH.

## Materials and methods

### Intracerebral hemorrhage mouse model

Male C57BL/6 mice (20–25 g, 8–10 week-old), purchased from the Chinese Academy of Military Sciences (Beijing, China), were housed in the animal room of the General Hospital of Tianjin Medical University for 1 week before the experiments, with a 12 h light/dark cycle and adequate food and water supply to acclimatize to the environment. All animal experiments were approved by the Animal Experiments Ethics Committee of Tianjin Medical University (approval number: IRB2012-028-02) and were performed in accordance with the Guide for the Care and Use of Laboratory Animals.

According to a previous study (Pan et al., 2018), the ICH model was induced by injection of collagenase type VII (C0773, Sigma-Aldrich, St. Louis, MO, United States) into the C57BL/6 mice. Briefly, the mice were anesthetized *via* intraperitoneal injection of a mixture of ketamine (100 mg/kg) and xylazine (10 mg/kg) (2:1 volume ratio), and then mounted on a stereotaxic frame (RWD Life Science, Shenzhen, China). A 10 mm surgical incision was made in the middle of the head to expose the skull. Using a micro-infusion pump (26-G; Hamilton, Manitowoc, WI, United States), 0.0375 U of collagenase type VII in 0.5 μL of 0.9% saline was stereotactically injected into the right basal ganglia (coordinates 0.2 mm anterior, 3.5 mm ventral and 2.5 mm lateral to the bregma) with a rate of 0.5 μL/min. After injection, the micro-infusion pump needle was left in place for 15 min and then slowly withdrawn at a rate of 1 mm/min. The incision was sutured and disinfected after the operation. Sham mice received the same procedures except for the collagenase infusion.

### Treatments

**FIGURE 1:**
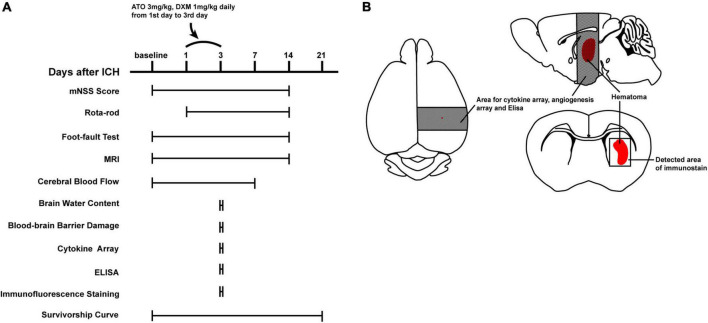
Experimental design. **(A)** Schematic of the experimental timeline for functional and pathological assessments. **(B)** Schematic of the brain of ICH mice. Dark shaded areas of brain tissue were used for cytokine arrays, angiogenesis arrays, and ELISA. Lightly shaded areas were used for the immunofluorescence microphotographs.

The mice (*n* = 150) were randomly divided into 5 groups: (1) sham group (normal mice, *n* = 20); (2) vehicle group (ICH mice, *n* = 35); (3) ATO group (*n* = 30), where the ICH mice were treated with ATO (PZ0001, Sigma-Aldrich, St. Louis, MO, United States); (4) DXM group (*n* = 30), where the ICH mice were treated with DXM (D1756, Sigma-Aldrich, St. Louis, MO, United States); (5) ATO-DXM group (*n* = 35), where the ICH mice were treated with both ATO and DXM. The dosage of ATO and DXM were 3 mg/kg and 1 mg/kg, respectively, based on earlier studies (Chen et al., 2003; Matsushita et al., 2014). Both the vehicle group and the sham group were treated with 0.9% saline as the placebo. After establishing the ICH model in 24 h, oral gavage of the corresponding treatment in each group was started and subsequent tests were performed according to the experimental design (Figure 1).

### Survival analysis

The survival analysis in this study was performed by Kaplan–Meier Curve to evaluate the rate at which protocols reached specific terminal events, stratified by intervention conditions. We observed the experimental mice for 20 days. Natural death was regarded as a terminal event, while manual execution was considered censored data.

### Behavioral tests

In this study, three behavioral tests, namely mNSS, foot-fault test, and Rota-rod test, were used to determine the functional recovery of mice after ICH. All of the above behavioral tests were monitored by three blinded investigators.

The mNSS consists of motor, sensory, cognition, reflex, and abnormal movement tests scored on a scale of 0–18 (Hua et al., 2002). The higher the score, the worse the neurological function. We recorded the mNSS for each group on days 1, 3, 7, and 14 after ICH to assess the neurological dysfunction.

For the assessment of forelimb dysfunction, mice were subjected to a foot-fault test on days 1, 3, 7, and 14. The mice were placed on a footstep grid, and the number of steps that left forelimb steps fell off the grid (faulty steps) and the number of steps that each forelimb passed through the grid (total steps) was recorded. The forelimb dysfunction rate was expressed as: forelimb dysfunction rate = faulty steps/total steps × 100%.

Motor coordination and learning ability of ICH mice were assessed at baseline time and on days 1, 3, 7, and 14 after ICH using the Rota-rod device (RWD Life Science, Shenzhen, China) (Xu et al., 2017). At baseline time, mice were placed on the Rota-rod device for three low-speeds (4 rpm/min) and four acceleration experiments (from 4 rpm/min to 40 rpm/min in 5 min) to allow the mice to acclimatize to the experimental environment. At the next experimental time points, each mouse received four acceleration speed experiments at 300 s every 30 min. The time of the first fall of the mice was recorded, and the passive motion after the Rota-rod rotation was also considered a fall.

### Magnetic resonance imaging

Hematoma volume changes in ICH mice were measured by an magnetic resonance imaging (MRI) scanner. MRI scanning was performed by a 3T scanner (MR750, GE Healthcare, Chicago, IL, United States) with a 6-cm internal diameter 4-channel phase-arrayed animal coil (Magtron, Shenzhen, China) at baseline and 1, 3, 7, and 14 days after ICH. Mice were anesthetized with a mixture of ketamine (100 mg/kg) and xylazine (10 mg/kg) (2:1 volume ratio, intraperitoneal injection) in a supine position with appropriate head immobilization to limit head movements. MR images from the frontal pole to the brainstem were acquired using a three-dimensional gradient recalled echo (3D-GRE) sequence with the following parameters: repetition time (TR)/effective echo time (TE), 200/6 ms; field of view (FOV), 18 × 18 mm; matrix, 512 × 512; slice thickness, 1.0 mm; and a number of slices, 13. Hematoma volume was calculated as in a previous study (Wang et al., 2018) by Horos (version3.3.3, The Horos Project). In all images with lesions, the hematoma area was outlined along the border and multiplied by the slice thickness. The hematoma volume obtained from all images was summed to obtain the total hematoma volume.

### Laser speckle imaging

Cerebral blood flow (CBF) was measured in mice at baseline and on days 1, 3, and 7 after ICH using a laser speckle contrast imager (PeriCam PSI, Perimed, Stockholm, Sweden). Briefly, mice were anesthetized and were mounted on a stereotaxic frame. After cleaning and disinfection, a 1 cm incision was made in the midline of the scalp to expose the skull for laser irradiation. The laser cross-center was aligned with the bregma and CBF was recorded for 2 min after stabilization. After the measurement, the incision was sutured, and the mouse was placed in a heated cage until awake. The 1-min region of interest (ROI) with stable CBF was selected and analyzed by PIMsoft (version 1.5.4.8078, Perimed, Stockholm, Sweden). The results were expressed as the CBF ratio of each modeling group to the sham group.

### Brain water content

The Brain water content was measured according to the wet/dry method (Li et al., 2017) on the 3rd day after ICH. After anesthesia, the mice were sacrificed, and their brains were quickly removed and obtained. After measuring the wet brain weights (WW) of the brains, they were dried at 100°C for 24 h to obtain the dry brain weights (DW). The brain water content was calculated according to the formula: brain water content = (WW - DW)/WW × 100%.

### Blood-brain barrier damage

The brain tissue Evans blue (EB) dye extravasation method was applied to determine the extent of blood-brain barrier (BBB) damage on the 3rd day after ICH. EB (E8010, Solarbio, Beijing, China) with a final concentration of 2% was administered intraperitoneally (4 ml/kg) in anesthetized mice and allowed to circulate *in vivo* for 3 h (Manaenko et al., 2011). After transcardiac perfusion with phosphate buffer saline (PBS, P1010, Solarbio, Beijing, China), the right hemisphere was collected and homogenized in a glass homogenizer with PBS (1,100 μL). The brain tissue homogenates were then sonicated and centrifuged (30 min, 15,000 *g*, 4°C). The resulting supernatant was collected and mixed with an equal amount of 50% trichloroacetic acid (T104261, Aladdin, Shanghai, China). After incubation overnight at 4°C, the mixture was centrifuged at 15,000 *g* for 30 min at 4°C. Finally, the absorbance of the supernatant at 610 nm was determined using a microplate reader. The extent of BBB damage in each group (vehicle, ATO, DXM, and ATO-DXM) was evaluated by comparing the experimental group to the OD_610_ of the sham group.

### Cytokine array

In this study, inflammation was detected by the Mouse Cytokine Array Panel A Kit (ARY006, R&D Systems, Minneapolis, MN, United States) on the 3rd day after ICH. A total of 40 inflammatory cytokines were assayed by the kit, such as Granulocyte-colony stimulating factor (G-CSF), Granulocyte macrophage-colony stimulating factor (GM-CSF), CXCL1, CXCL9, CXCL11, CXCL12, CXCL13, C5a, Soluble intercellular adhesion molecule-1 (sICAM-1), CCL1, CCL5, CCL13, CCL17, Interferon-gamma (IFN-γ), IL-1ra, IL-1α, IL-1β, IL-2, IL-3, IL-4, IL-5, IL-6, IL-7, IL-10, IL-13, IL-12p70, IL-16, IL-17, IL-23, IL-27, Inducible protein-10 (IP-10), Macrophage-colony stimulating factor (M-CSF), Monocyte chemotactic protein-1 (MCP-1), MCP-5, Macrophage inflammatory protein-1α (MIP-1α), MIP-1β, MIP-2, Tissue inhibitor of matrix metalloprotease-1 (TIMP-1), Tumor necrosis factor-a (TNF-a), and Triggering receptor expressed on myeloid cells-1 (TREM-1).

After transcardial perfusion, brain tissue (130 mg) surrounding the around the right hemisphere (including the hematoma) was obtained and homogenized with protease inhibitors. Triton X-100 (T9284, Sigma-Aldrich, St. Louis, MO, United States) was added to the brain tissue homogenate at a concentration of 1% and stored frozen at −80°C overnight. After thawing the next day, the brain tissue homogenate was centrifuged at 10,000 *g* at 4°C for 5 min, to remove cellular debris. The supernatant of the brain tissue homogenate was further collected, and the total protein concentration was quantified using a bicinchoninic acid (BCA) assay (Bicinchoninic Acid Protein Assay Kit; 23227, Thermo Fisher Scientific, Waltham, MA, United States) according to the manufacturer’s instructions. Background density in each group was removed to reduce the inter-array variations, and cytokine expression was expressed as mean density. The mean density of each cytokine in the sham group was chosen as the standard to analyze the relative expression of cytokine in the other 4 groups. All of the above image processing was done by Image J (version 1.52a, NIH, Bethesda, MD, United States).

### Enzyme-linked immunosorbent assay

The oxidative and antioxidative effects were detected using the enzyme-linked immunosorbent assay (ELISA) kit in each group on the 3rd day after ICH. The brain tissue homogenate supernatant was obtained as described above and subsequent experimental protocols followed the manufacturer’s instructions. Oxidative accumulation was determined by measuring the content of lipid peroxide (Malondialdehyde, MDA; A003-1, Jiancheng Bioengineering Institute, Nanjing, China) in the supernatant of brain tissue homogenate. Oxygen radical activity was measured by the enzymatic activity of Super Oxide Dismutase (SOD; A001-1, Jiancheng Bioengineering Institute, Nanjing, China) and Catalase (CAT; A007-1, Jiancheng Bioengineering Institute, Nanjing, China). The lower MDA content (nmol/mg protein), or the higher SOD (U/mg protein) and CAT (U/mg protein) activity indicated a better treatment effect in this group.

### Immunofluorescence staining

Immunofluorescence staining was performed on the obtained brain tissue. Briefly, after fixing the obtained brain tissue with paraformaldehyde for 24 h, the tissue was dehydrated and cleared, and further embedded in paraffin and sectioned consecutively in the coronal plane with a thickness of 7 μm. The paraffin sections were dewaxed and dehydrated and boiled in boiling citrate buffer (C1010, Solarbio, Beijing, China) for 30 min to repair antigens. Naturally cooled sections were washed three times in PBS. 0.3% Triton X-100 was used to rupture the membranes and 5% bovine serum albumin (BSA; B2064, Sigma-Aldrich, St. Louis, MO, United States) was applied to block non-specific binding at 37°C for 1 h. The sections were incubated with rabbit anti-NeuN (neuronal nuclei) antibody (1:300; ab177487, Abcam, Cambridge, United Kingdom) overnight at 4°C, and then with donkey anti-Rabbit IgG (H + L) (2 μg/mL; A21207, Thermo Fisher Scientific, Waltham, MA, United States) for counterstaining. Then the sections were stained using a TUNEL (terminal deoxynucleotidyl transferase-dUTP nick end labeling) staining kit (G3250, Promega, Madison, WI, United States). Finally, the nuclei was stained by 4’,6-diamidino-2-phenylindole (DAPI; ab104139, Abcam, Cambridge, United Kingdom). The neuronal apoptosis and proliferation surrounding the hematoma were observed using a fluorescence microscope (BX53, Olympus, Tokyo, Japan). Three sections with six fields of view per section were observed in each group. Results were expressed as the percentage of proliferation-positive neurons (Ki67 + NeuN double-stained cells/NeuN stained cells) and the percentage of apoptotic-positive neurons (TUNEL + NeuN double-stained cells/NeuN stained cells).

### Statistical analysis

All data analysts were blinded to the grouping and treatment. In the present study, statistical analysis was performed by SPSS (version 25.0, IBM, Armonk, NY, United States), and image analysis was performed using Image J (Version 1.52a, NIH, Bethesda, MD, United States). ELISA results were analyzed with the non-parametric test, and other data were analyzed with a one-way analysis of variance (ANOVA) with the Bonferroni *post hoc* test. The survival curve was produced with GraphPad Prism 9 (version 9.0, GraphPad Software, San Diego, CA, United States), and the HR and 95% CI were analyzed by SPSS. All data were expressed as mean ± SEM. *P*-values less than 0.05 were considered statistically significant.

## Results

### ATO-DXM treatment improves survival rate in intracerebral hemorrhage mice

**FIGURE 2:**
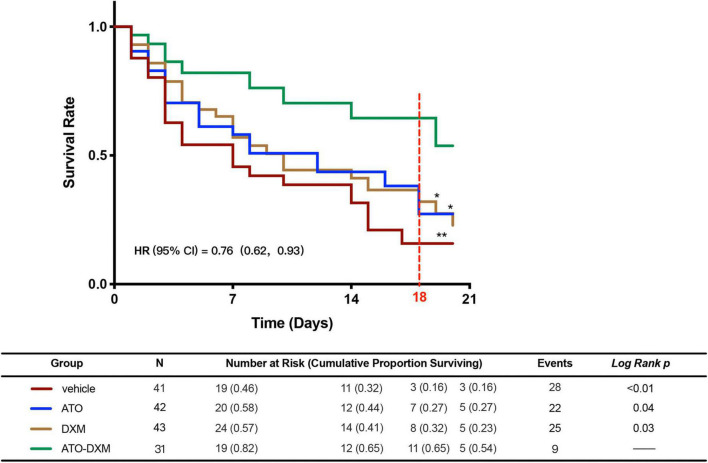
ATO-DXM treatment improves the survival rate of ICH mice. Kaplan–Meier Survival Curves were plotted for each group of ICH mice. Compared to the vehicle group, ATO treatment, DXM treatment, and ATO-DXM treatment significantly increased the survival rate. Compared to monotherapy, the combination of ATO and DXM treatment considerably improved the survival rate at day 18 after ICH. *Log-Rank p < 0.05; **Log-Rank p < 0.01.

After successful modeling of the ICH in mice, survival statistics were performed for 20 days in the vehicle group, ATO group, DXM group, and the ATO-DXM group. According to the plotted survival curves (Figure 2), the survival prognosis of the vehicle group was the worst, while the survival curves of the ATO-DXM group were statistically significantly different from other treatment groups, with log-rank *p* < 0.01 vs the vehicle group, with log-rank *p* = 0.04 vs the ATO group, with log-rank *p* = 0.03 vs the DXM group. The risk ratio (HR) of the ATO-DXM group was 0.76 with a 95% CI of 0.62–0.93, suggesting that the ATO-DXM group had the best survival prognosis. Meanwhile, we plotted the risk table and performed survival analysis in the vehicle group (*n* = 41), the ATO group (*n* = 42), the DXM group (*n* = 43), and the ATO-DXM group (*n* = 31) and counted the number of mice at risk and the cumulative proportion surviving. It can be seen that on the seventh day the number at risk of the vehicle group was 19 and the cumulative survival rate was 0.46, the number at risk of the ATO group was 20 and the cumulative survival rate was 0.58, the number at risk of the DXM group was 24 and the cumulative survival rate was 0.57, and the number at risk was 19 and the cumulative survival rate was 0.82. On day 14, the number at risk for the vehicle group 14 was 11, with a cumulative survival rate of 0.32, the number at risk for the ATO group was 12, with a cumulative survival rate of 0.44, and the number at risk for the DXM group was 14, with a cumulative survival rate of 0.41, and the number at risk for the ATO-DXM group was 12 in the ATO-DXM group, and the cumulative survival rate was 0.65. On day 21, the number at risk of the vehicle group was 3 with a cumulative survival rate of 0.16, the number at risk for the ATO group was 5 with a cumulative survival rate was 0.27, and the number at risk for the DXM group was 5 with cumulative survival rate was 0.23, and the number at risk for ATO-DXM group was 5 with cumulative survival rate 0.54. In addition, we noticed that the cumulative survival rate of the ATO-DXM group on day 18 reached the maximum value relative to the cumulative survival rate of the remaining three groups (marked with a red dotted line). On day 18, the number at risk of the vehicle group was 3 and the cumulative survival rate was 0.16, the number at risk of the ATO group was 7 and the cumulative survival rate was 0.27, the number at risk of the DXM group was 8 and the cumulative survival rate was 0.32, and the number at risk of ATO-DXM group was 11 and the cumulative survival rate was 0.65. The prognostic results for the survival of mice in each group after ICH were counted, and the deaths were 28 in the vehicle group, 22 in the ATO group, 25 in the DXM group, and only 9 in the ATO-DXM group.

### ATO-DXM treatment accelerates neurological recovery after intracerebral hemorrhage

**FIGURE 3:**
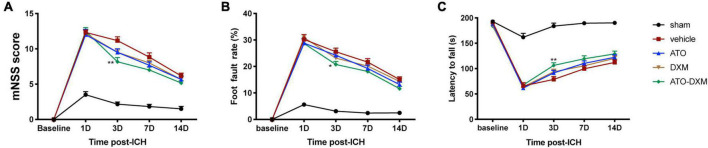
Neurological functional evaluations. **(A)** mNSS after ICH; **(B)** foot-fault test after ICH; **(C)** Rota-rod test after ICH. *p < 0.05, **p < 0.01 vs vehicle group.

We used the mNSS score to evaluate the neurological function of ICH mice and found that all mice received high scores on the 1st day after ICH, indicating that ICH caused significant neurological impairment. As expected, the mNSS score gradually decreased in each treatment group from day 3–14 compared to the sham group, suggesting recovery of neurological function due to the treatment. We noted a significant decrease in mNSS scores on day 3 after ICH for ATO-DXM treatment compared with a decrease in mNSS scores for ATO monotherapy or DXM monotherapy. Thus, the combination of ATO and DXM treatment possessed the fastest recovery of neurological function (Figure 3A).

Similarly, we used the foot-fault test to evaluate limb dyskinesia after ICH by analyzing the percentage of fault steps. Compared to the sham group, the motor function of the mice was improved on the 3rd day after treatment, as indicated by decreased foot fault rate (Figure 3B). Again, we found that the combination of ATO and DXM treatment had better therapeutic effects compared to ATO monotherapy or DXM monotherapy since the foot fault rate of mice in the ATO-DXM group decreased significantly compared to the vehicle group.

Lastly, we used the Rota-rod test to evaluate the neurological prognosis of mice by counting the time they move on the Rota-rod device. Significant motor function improvement was observed in the ATO-DXM group on day 3 after ICH compared with the vehicle group (Figure 3C). However, monotherapy by ATO or DXM had no significant motor function improvements compared to the vehicle group, which was consistent with the mNSS test and foot-step test.

Clearly, a therapeutic effect had been achieved in the ATO-DXM group rather than in any other monotherapeutic one during days 3–7. However, the difference in therapeutic effect among all the above neurofunctional tests disappeared during days 7–14. It strongly indicates that the therapeutic effects of this combined strategy didn’t persist with medicine withdrawal. In order to elucidate the potential mechanisms underlying the dominance of ATO-DXM treatment on intracerebral hemorrhage, hematoma absorption, cerebral blood flow, inflammation cytokines, oxidative production, and cell death and proliferation were all measured on the 3rd day of treatment after ICH, the only time point at which there was significant neurological recovery.

### ATO-DXM treatment reduces hematoma volume

**FIGURE 4:**
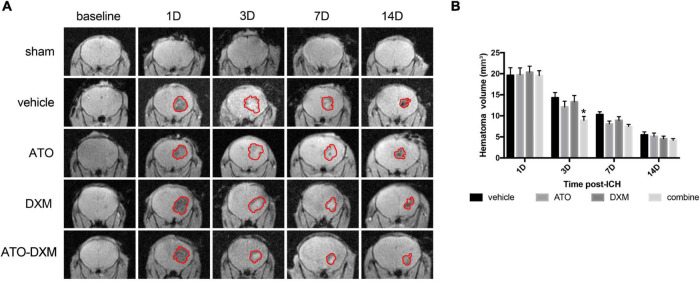
Dynamic changes in hematoma volumes were estimated by 3D-GRE MRI. **(A)** Representative 3D-GRE MRI images (coronal sections) at baseline and 1, 3, 7, and 14 days after ICH. The areas circled in red indicate the hematoma. **(B)** Quantification of total hematoma volume at baseline, 1, 3, 7, and 14 days after ICH. *p < 0.05 vs vehicle group.

Intracranial conditions of the ICH mice were visualized using a 3D-GRE sequence of MRI. The intracranial hematoma showed hypointense on MRI images on day 1 and day 14 (Figure 4A). On days 3 and 7, it showed hyperintensity, which was believed to be attributed to the presence of extracellular methemoglobin (Karki et al., 2009). We selected mice with no significant difference in hematoma volume on day 1 after ICH and continued with the subsequent MRI studies. The result showed that the hematoma volume began to decrease from day 3 in each treatment group. The results of hematoma absorption were consistent with the results of neurological recovery: only the ATO-DXM group showed a significant reduction in hematoma on day 3 compared to the vehicle group, with no significant improvement after day 3 (Figure 4B). Thus, the efficiency of hematoma elimination in the ATO-DXM treated ICH mice decreased rapidly with medicine discontinuation.

### Cerebral blood flow and brain edema

**FIGURE 5:**
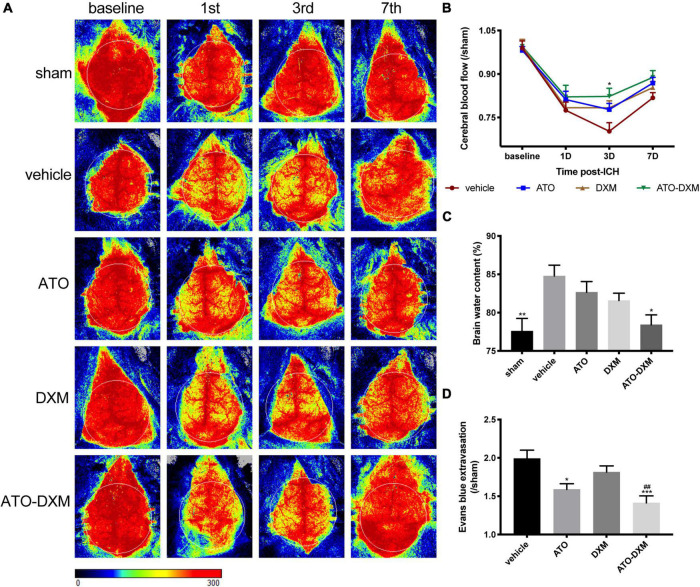
Evaluation of CBF, brain edema, and BBB integrity after ICH. **(A)** Representative images of CBF changes at baseline, 1, 3, and 7 days after ICH for each group. **(B)** Trend and statistical comparison of CBF in each group after ICH. Results were expressed as a ratio of each group compared to the sham group. **(C)** Brain water content on day 3 after ICH. **(D)** BBB integrity was evaluated by Evans blue extravasation on day 3 after ICH. *p < 0.05, **p < 0.01, ***p < 0.001 vs. vehicle group. ##*p* < 0.01 vs DXM group.

Cerebral blood flow (CBF) was measured at baseline, day 1, 3, and 7 after ICH using a laser speckle contrast imager, and changes in each group were dynamically analyzed. The representative images suggested that CBF was significantly reduced after injury and reperfusion started on day 3 (Figure 5A). Meanwhile, we found that the ATO-DXM group showed positive CBF improvement on day 3 compared to the vehicle group, while the ATO group and DXM group failed to show improvements (Figure 5B).

Brain water content detection, the classic method for measuring brain edema, revealed that ICH caused significant brain edema. Compared to the sham group, brain water content increased by 7.1% in the vehicle group, 5.2% in the ATO group, 3.4% in the DXM group, and 1.3% in the ATO-DXM group. Thus, combined ATO and DXM treatment significantly reduced the degree of brain edema on the 3rd day after ICH (Figure 5C).

After 3 consecutive days of medicine administration following ICH, we performed the EB extravasation test and found that the mice in the ATO-DXM had improved BBB function. We noticed that the efficacy of DXM in the treatment of BBB functional defects did not have a significant advantage, while improved BBB function was observed in the ATO group when compared to the vehicle group. Moreover, the combination between DXM and ATO had an obvious improvement compared to the DXM group and the vehicle group.

### Expression of inflammatory cytokines

**FIGURE 6:**
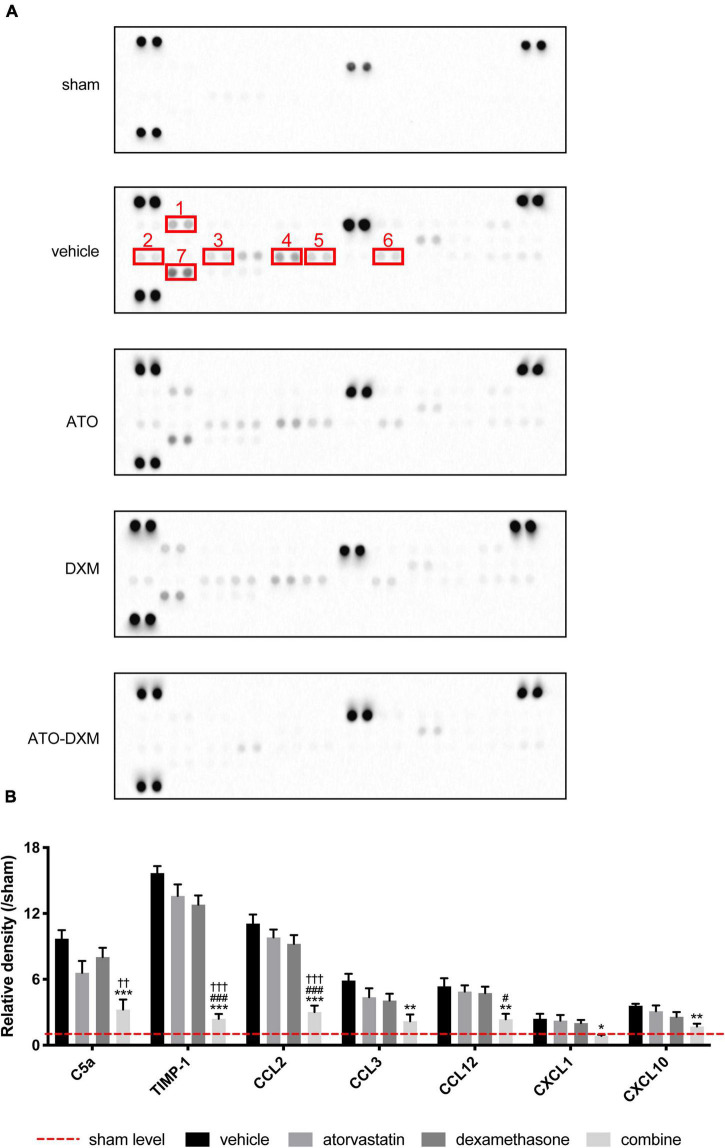
Inflammatory cytokines in perihematomal tissues detected by cytokine array for 3 consecutive days after ICH. **(A)** Expression of inflammatory cytokines in each group, 1–7 representing C5a, CXCL10, CXCL1, CCL2, CCL12, CCL3, and TIMP-1, respectively. Each cytokine in a cytokine array was presented in duplicate, indicated by two adjacent black dots. **(B)** Statistical comparisons of inflammatory cytokines expressed as the relative density of inflammatory cytokine density of interest/corresponding inflammatory cytokine density in the sham group. *p < 0.05, **p < 0.01, ***p < 0.001 vs vehicle group; #*p* < 0.05, ###*p* < 0.001 vs ATO group; ††*p* < 0.01, † † †*p* < 0.01 vs DXM group.

After treating the ICH mice for 3 days, we examined the expression of various inflammatory cytokines by cytokine array. We found that inflammatory cytokines expression was lower in the ATO-DXM group and angiogenic cytokines expression was much stronger in the ATO-DXM group and ATO group (Figure 6A).

After quantifying the expression of inflammatory factors, the data showed that 7 of the 40 inflammatory factors C5a, TIMP-1, CCL2, CCL3, CCL12, CXCL1, and CXCL10) showed significant differences between groups (Figure 6B). Although C5a expression in the ATO-DXM group didn’t differ from that in the ATO group, it was significantly lower than that in the vehicle group and DXM group. The effect of ATO or DXM monotherapy on reducing the C5a levels was not obvious. The expression levels of TIMP-1 and CCL2 were significantly lower in the ATO-DXM group compared with other groups, whereas neither the ATO group nor DXM group had any difference compared with the vehicle group. CCL3 expression in the ATO-DXM group was only significantly decreased relative to the vehicle group, while neither the ATO group nor DXM group showed any differences from the vehicle group. CCL12 level in the ATO-DXM group was significantly lower than that in the vehicle group and ATO group. As for the ATO group and DXM group, both of them failed to show significant differences relative to the vehicle group. CXCL1 inflammatory factor test results showed that the expression level of the ATO-DXM group was lower than that of the vehicle group, while ATO and DXM monotherapy on reducing CXCL1 was not obvious. In the comparison of the CXCL10 expression level, the ATO-DXM group was only significantly lower than that of the vehicle group, while the ATO group and DXM group did not show significant differences from the vehicle group. Thus, ATO combined with DXM significantly inhibited the expression of the inflammation-related cytokines when compared with the monotherapeutic groups.

### Oxidative and antioxidant effects

**FIGURE 7:**
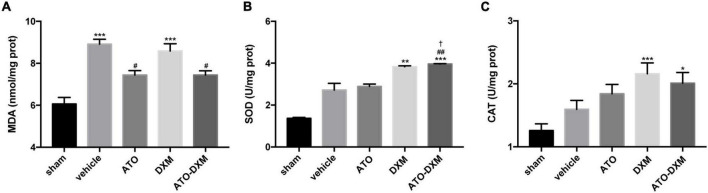
Assessment of oxidative and antioxidative capacities of the tissue surrounding the hematoma on day 3 after ICH. **(A)** MDA content. **(B)** SOD activity. **(C)** CAT activity. *p < 0.05, **p < 0.01, ***p < 0.001 vs sham group; #*p* < 0.05, ##*p* < 0.01 vs vehicle group; †*p* < 0.05 vs ATO group.

To access the oxidative accumulation and anti-oxidation effects on hematoma, we selected two antioxidant enzymes, SOD and CAT, and lipid peroxide MDA. On the 3rd day after ICH, MDA contents increased in each ICH group compared with the sham group, with a significant increase in the vehicle group and DXM group. Meanwhile, after 3 consecutive days of treatment, the MDA levels in both the ATO and the ATO-DXM groups were significantly lower than those in the vehicle and DXM groups, indicating that ATO and ATO-DXM had an excellent therapeutic effect on reducing the MDA content (Figure 7A). As for the activity of SOD antioxidant enzymes, the results were as follows. On day 3, the SOD activity of both DXM and ATO-DXM groups was significantly improved compared to the sham group. It showed a clear advantage over the vehicle group. It was worth mentioning that the SOD activity of the ATO-DXM group on the 3rd day also was significantly higher than that of the ATO group, indicating that the ATO-DXM combination had a better therapeutic effect over the ATO only (Figure 7B). As for CAT, although the DXM group and the ATO-DXM group had significant improvement over the sham group on the 3rd day, the therapeutic effect of ATO and DXM monotherapy was not significant compared with the vehicle group (Figure 7C).

### Neuronal proliferation and apoptosis

**FIGURE 8:**
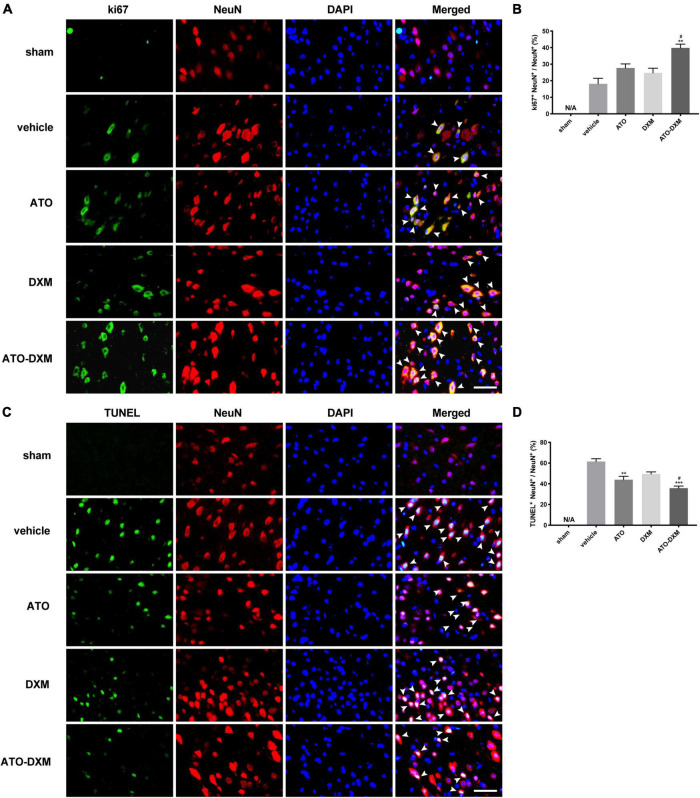
Immunofluorescence co-staining of neuronal proliferation and apoptosis from the perihematomal on day 3 after ICH. **(A)** Co-staining of neuronal proliferation. Fluorescent colors: ki-67, green; NeuN, red; nuclei, blue. Proliferating cells, indicated by arrows, were counted under a fluorescence microscope (original magnification, 400 × ; scale bar, 200 μm). **(B)** Statistical comparisons of Ki67 + neuronal cells. **(C)** Co-staining of neuronal apoptosis. Fluorescent colors: TUNEL, green; NeuN, red; nuclei: blue. Apoptotic cells, indicated by arrows, were counted under a fluorescence microscope (original magnification, 400 × ; scale bar, 200 μm). **(D)** Statistical results of TUNEL + neuronal cells. *p < 0.05, **p < 0.01 vs vehicle group; #*p* < 0.05 vs DXM group.

Representative fluorescent images of neuronal proliferation from the perihematomal showed immunofluorescence co-staining of ki67 (cell proliferation-associated marker, green), NeuN (neuron marker, red), and DAPI (nuclei marker, blue) on the 3rd day (Figure 8A). Quantitatively analysis showed that the ATO-DXM group presented a high percentage of proliferation-positive neurons, significantly higher than the vehicle group and the DXM group (Figure 8B). Although proliferation-positive neurons were presented in the vehicle group, the ATO group, and the DXM group, there were no significant differences between the three groups.

TUNEL (cell apoptosis-associated marker, green), NeuN (neuron marker, red), and DAPI (nuclei marker, blue) were co-stained to compare neuronal apoptosis in each group on the 3rd day (Figure 8C). There were no TUNEL^+^ cells in the sham group, and significant TUNEL^+^ apoptotic cells appeared in the other four groups. By statistical analysis, the opposite result was observed in neuronal apoptosis compared to neuronal proliferation (Figure 8D). The ATO-DXM group had the lowest rate of neuronal apoptosis, which was significantly lower than the vehicle and DXM groups on the 3rd day. In contrast to the ineffectiveness of neuronal proliferation, ATO monotherapy presented a significant reduction of neuronal apoptosis on the 3rd day. However, there was still no difference in the DXM group compared to the vehicle group.

## Discussion

In the present study, we used ATO and low-dose DXM to treat ICH mice and found that the combination of ATO and DXM significantly reduced the overall mortality of ICH mice. The combination exerted a synergistic effect of promoting the absorption of hematoma, alleviating cerebral inflammation, and reducing brain edema by activating multiple mechanisms, including reducing apoptosis and oxidative stress, and increasing cerebral blood flow. Thus, the combined treatment enhances neuroprotection, reduces secondary brain injury, and improves the prognosis of ICH. Compared to monotherapy by ATO or low-dose DXM, the combined therapy enhances the anti-inflammatory, anti-oxidative, and earlier neuroprotective effect in the acute stage of ICH.

Past studies on ICH mainly focused on two areas: one is to evaluate the value of neurosurgical interventions and post-onset hemostatic therapy such as FAST, SPOTLIGHT, and STOP-IT (Steiner et al., 2011; Gladstone et al., 2019); the other is to regulate the medication at different stages of ICH, including blood controlling, dehydration for the brain edema, and alleviating inflammation by steroids (Toyoda et al., 2021). The common objective of these studies is to achieve the goal of reducing the volume of hemorrhage after its onset or reducing the risk of hematoma enlargement in ICH patients. Unlike the former research, the present study is expected to interfere with the mechanism of the secondary brain injury, thereby alleviating brain edema and inflammation while improving the survival of ICH.

It is well recognized that inflammation triggered during the acute phase of hemorrhagic stroke is the main cause of secondary brain injury (Zheng et al., 2016). ATO and DXM play different roles in this process. Oral administration of ATO promotes safe and effective absorption of chronic subdural hematoma (CSDH) (Jiang et al., 2018), and ATO has been reported to regulate intracranial inflammatory response and reduce neurological damage (Rodriguez-Perea et al., 2017; Endres et al., 2018; Saliba et al., 2018). Moreover, DXM takes a stronger role than ATO in restraining inflammation and brain edema, and low dose DXM may avoid its adverse effects, including hemorrhagic tendency and endocrinopathy (Yang J. T. et al., 2011; Zaganas et al., 2011), thus the combination of ATO and DXM could further benefit CSDH patients *via* a synergistic anti-inflammatory effect (Wang et al., 2020).

The efficacy of statins for the treatment of stroke is controversial. Statins are used as lipid-lowering drugs clinically, and hypolipidemia is considered to be a risk factor for hemorrhagic stroke; dosage of statins in stroke also often leads to different outcomes. However, recently studies have shown that the lipid-lowering effect of statins is not related to the risk of ICH (Spence, 2020; Yu et al., 2022), and low-dose statin can produce a favorable prognosis (Chen et al., 2003; Amarenco et al., 2006; Biffi et al., 2011). Therefore, our study suggested that the short-term, low-dose medication by ATO/DXM could be effective in the acute stage of ICH onset. ICH impaired CBF and BBB function and caused brain edema on the 3rd day (Figure 4). The combination of ATO-DXM improved the BBB function and CBF reperfusion during a brief 3-day treatment instead of monotherapy (Figures 4C,D). In addition to its hypolipidemic effect, ATO reduces the levels of nitric oxide synthase (iNOS) and myeloperoxidase (MPO) in brain tissue surrounding the hematoma after ICH and reduces apoptosis, which is associated with inhibition of cerebral inflammation and repair of neurological function (Jung et al., 2004; Karki et al., 2009). Compared to DXM, ATO showed better regulation of apoptosis, cerebral blood flow, oxidative response, and BBB function.

The combination therapy reduced the activation of chemokine family members, including C5a, CXCL1, CXCL10, CCL2, CCL3, CCL12, and TIMP-1 (Figure 5). Among them, C5a (Khan et al., 2018), CXCL1, CXCL10 (Chan et al., 2021), CCL2, CCL12 (Ondondo et al., 2015), CCL3 (Zhang et al., 2020), TIMP-1 (Haeberle et al., 2017) are associated with inflammatory suppressive Treg, which functions to reduce brain edema and inflammatory infiltration. ATO can inhibit the inflammatory response and accelerate hematoma resorption by enhancing Treg cells in the brain, a specific type of immunosuppressive T cell that is considered to be an important negative regulator of inflammation, and can also activate Treg, improves hematoma clearance, and facilitates neurological recovery (Quan et al., 2019). Treg acts by reducing the transition of pro-inflammatory cells to the lesion site, and the imbalance of Treg and CD4 + IL-6 + T cells contributes to various vascular diseases (Mahmoud and Al-Ozairi, 2013). IL-6 and IL-8 at the lesion site accelerate the chemotaxis of Treg, which reduces the levels of IL-1/IL-6/IL-8 and TNF-α produced by inflammatory cells and secretes IL-10 and IL-13 to enhance anti-inflammatory effects (Pan et al., 2018). Furthermore, intracranial meningeal lymphatic vessels enhance drainage of acute subdural hematoma, whereas hemorrhage impairs drainage efficacy and lymphangiogenesis (Liu et al., 2020).

Oxidative stress caused by the toxic effects of reactive oxygen species (peroxides, superoxides, free radicals), is closely related to the inflammatory response and cell apoptosis (Yi et al., 2017). Our results demonstrated that oxidative toxicants could also be more excreted by combination therapy, confirming the important role of the medication in the clearance process of ICH: reducing oxidative stress and inflammatory responses, and inhibiting neuronal apoptosis. This study also showed the advantage of combination therapy in promoting neuronal proliferation, while the exact mechanism by which combination therapy promotes intracranial hematoma absorption remains elusive. On the one hand, we believe that neuronal proliferation is involved. Neuronal proliferation, an essential brain repair process after ICH, provides cytoprotective or trophic factors to cells surrounding hematoma, thereby achieving the purpose of clearing the hematoma and promoting the recovery of ICH (Shen et al., 2008; Zhou et al., 2018). On the other hand, activation of PPARγ may also be involved. Studies have been pointed out that simvastatin can promote hematoma clearance by activating PPARγ, enhancing the up-regulation of CD36 in microglia/macrophages, leading to M2 polarization of microglia, and promoting endogenous phagocytosis of erythrocytes (Wang et al., 2018). Further investigation of combination therapy mechanism on ICH hematoma clearance is necessary.

It is worth noting that the duration of ATO and DXM treatment is set to 3 days. On the one hand, in a trial administration of ATO, we found that there is no significant difference in mNSS after 3 or 7 days of treatment. On the other hand, considering that we aimed to study the treatment of low-dose stain in the acute phase of ICH, a short course of 3-day treatment was chosen. Lastly, DXM, as a hormone, should be administered as short a time as possible. However, the significant neuroprotection resulting from the combined treatment of ATO and DXM for 3 days suggests that this neuroprotective strategy is a potential adjunctive treatment for acute brain injury. There may be several reasons. Firstly, the *in situ* inflammation of ICH is always evoked as early as 0.5 h later but could be extended to 10 days (Gyoneva and Ransohoff, 2015). It indicates that therapy should be extended to 10 days at least. Though DXM persists for the longest half-time of a series of cortisones, it could not reduce apoptosis and modulate brain edema. In addition, either DXM or ATO could weakly modulate cytokines. Secondly, in addition to inflammation, apoptosis, BBB injury, cerebral blood flow, and oxidative product, the underlying mechanisms of secondary brain injury in ICH are unknown and still under investigation. For example, the disturbances in the lymphatic drainage system of the injured brain and the function of the different inflammatory cells are responsible for ICH brain injury, but some of these factors were weakly regulated by the combined treatment.

Another noteworthy point is that most of our studies showed the benefit of combined therapy was at day 3, while the greatest difference in cumulative survival rate occurred at day 18, which we believe is related to secondary brain injury. Secondary brain injury after ICH is for the most part due to the presence of intraparenchymal blood, which leads to the destruction of the blood-brain barrier, brain edema and brain cell death through inflammatory reaction, oxidative stress, cytotoxicity and other pathological pathways (Aronowski and Zhao, 2011). These pathological processes are motor events that perpetuate dysfunction over time (Gyoneva and Ransohoff, 2015; Li et al., 2020). Studies have shown that inflammatory responses to ICH occur as early as 15 min after the onset of ICH, with activation of inflammatory cytokines in the ICH affected hemisphere reaching maximum between 1 and 3 days and remaining elevated for weeks (Zhao et al., 2007). Therefore, early combined pharmacological intervention in ICH improved prognosis by accelerating hematoma clearance and controlling the development of secondary brain injury. However, the monotherapy and vehicle groups failed to effectively control early secondary brain injury. Secondary brain injury persisted and mortality remained high, ultimately resulting in the largest difference in cumulative survival rate at day 18. Further research on time points for ICH treatment may reveal more meaningful findings.

## Conclusion

In conclusion, in an established murine ICH model, we demonstrated the therapeutic potential of a short-term synergistic combination of ATO and low-dose DXM in improving post-ICH functional recovery and survival, possibly through an inhibitory modulation of the post-ICH inflammatory response. Future dose-response studies, as well as studies regarding the optimal time window for intervention, are warranted to validate the preclinical efficacy of this drug combination for the treatment of ICH.

## Data availability statement

The original contributions presented in this study are included in the article/supplementary material, further inquiries can be directed to the corresponding author/s.

## Ethics statement

The animal study was reviewed and approved by the Animal Experiments Ethics Committee of Tianjin Medical University.

## Author contributions

YS, JH, and WQ: study design. YS and XL: experiment implementation. JY, ZS, WJ, and ML: data analysis. YS, XZ, and WQ: manuscript writing. YQ, CG, ZG, HL, JH, and RJ: technical support. RJ and WQ: material support and supervision. All authors contributed to the article and approved the submitted version.
